# Systemic Health Effects of Oleuropein and Hydroxytyrosol Supplementation: A Systematic Review of Randomized Controlled Trials

**DOI:** 10.3390/antiox13091040

**Published:** 2024-08-27

**Authors:** Oleg Frumuzachi, Laura Ioana Gavrilaș, Dan Cristian Vodnar, Sascha Rohn, Andrei Mocan

**Affiliations:** 1 Department of Pharmaceutical Botany, Faculty of Pharmacy, “Iuliu Hațieganu” University of Medicine and Pharmacy, 23 Gheorghe Marinescu Street, 400337 Cluj-Napoca, Romania; oleg.frumuzachi@elearn.umfcluj.ro (O.F.); amocanm@gmail.com (A.M.); 2 Department of Food Chemistry and Analysis, Institute of Food Technology and Food Chemistry, Technische Universität Berlin, Gustav-Meyer-Allee 25, 13355 Berlin, Germany; 3 Department of Bromatology, Hygiene, Nutrition, “Iuliu Hațieganu” University of Medicine and Pharmacy, 23 Gheorghe Marinescu Street, 400337 Cluj-Napoca, Romania; laura.gavrilas@umfcluj.ro; 4 Department of Food Science, University of Agricultural Sciences and Veterinary Medicine, 3-5 Mănăştur Street, 400372 Cluj-Napoca, Romania; dan.vodnar@usamvcluj.ro

**Keywords:** Mediterranean diet, antihyperlipidemic activity, *Olea europaea* L., secoiridoids, 3,4-dihydroxyphenylethanol

## Abstract

Non-communicable diseases (NCDs) cause 41 million deaths annually, accounting for 74% of global fatalities. The so-called Mediterranean diet, with its especially significant consumption of olive oil, has shown promising results in reducing the risk of developing NCDs, such as cardiovascular, liver, or bone diseases. In the context of the nutritional health benefits of foods, phenolic compounds such as olive oil’s main components, oleuropein (OLE) and hydroxytyrosol (HT), have been shown to possess different beneficial effects. However, no systematic review has evaluated the health-promoting effects of OLE and HT until now. Consequently, this systematic review analyzed 12 human randomized controlled trials (RCTs), involving 683 participants, to assess the effects of supplements, pure compounds, or enriched foods containing OLE and HT regarding systemic health outcomes, including CVD risk factors, liver parameters, and bone, joint, and cognitive health. The review found contrasting but encouraging results, with some studies reporting significant modulation of body weight, lipid profile, and glucose metabolism, and improvements in bone, joint, and cognitive functions. The studies described different dosages and forms of supplementation, ranging from 5 mg/d HT to 990 mL/d olive leaf infusion (320.8 mg OLE and 11.9 mg HT), highlighting the need for further research to determine the optimal dosing and duration. Despite the mixed outcomes, OLE and HT supplementation show potential for improving some of the cardiometabolic health outcomes and bone, joint, and cognitive health. However, further studies are necessary to understand their benefits better and address existing limitations.

## 1. Introduction

Non-communicable diseases, such as cardiovascular diseases, diabetes, cancers, and chronic respiratory ones, represent a significant global health challenge, causing the death of 41 million people annually, accounting for 74% of all global fatalities [1]. In 2019, approximately 17.9 million individuals died because of cardiovascular diseases (CVDs), representing roughly 32% of the total global mortality. Among these fatalities, around 16 million were attributed to heart attacks and strokes [2]. The risk factors for CVDs include dyslipidemia, high blood pressure, hyperglycemia, obesity, physical inactivity, and unhealthy dietary habits [3]. Epidemiological studies have shown that the so-called Mediterranean diet (MD) positively influences cardiometabolic health and helps prevent primary CVD events [4]. The MD usually consists of omega-3 polyunsaturated fats, such as fish; unsaturated fats, such as olive oil; fruits and vegetables, such as tomatoes, garlic, and green leafy vegetables; whole grain foods that are high in fiber, such as whole wheat, barley, and oats; and nuts and legumes, such as peanuts, almonds, sesame and flaxseeds [5]. 

A systematic review and meta-analysis of prospective studies and randomized controlled trials (RCTs) found that individuals with higher adherence to the MD had a lower incidence of CVD and reduced cardiovascular mortality [6]. The beneficial effects on CVD outcomes were attributed to consuming fruits, vegetables, and legumes, and especially significant was the application of olive oil to almost all recipes [7]. Regarding these, a meta-analysis conducted by Martínez-González et al. revealed inverse associations between olive oil intake and CVD events, particularly stroke [8]. Additionally, a further meta-analysis also conducted by Martínez-González reported a 16% reduced risk of CVD for every 25 g/d increase in olive oil consumption [9]. 

**Figure 1:**
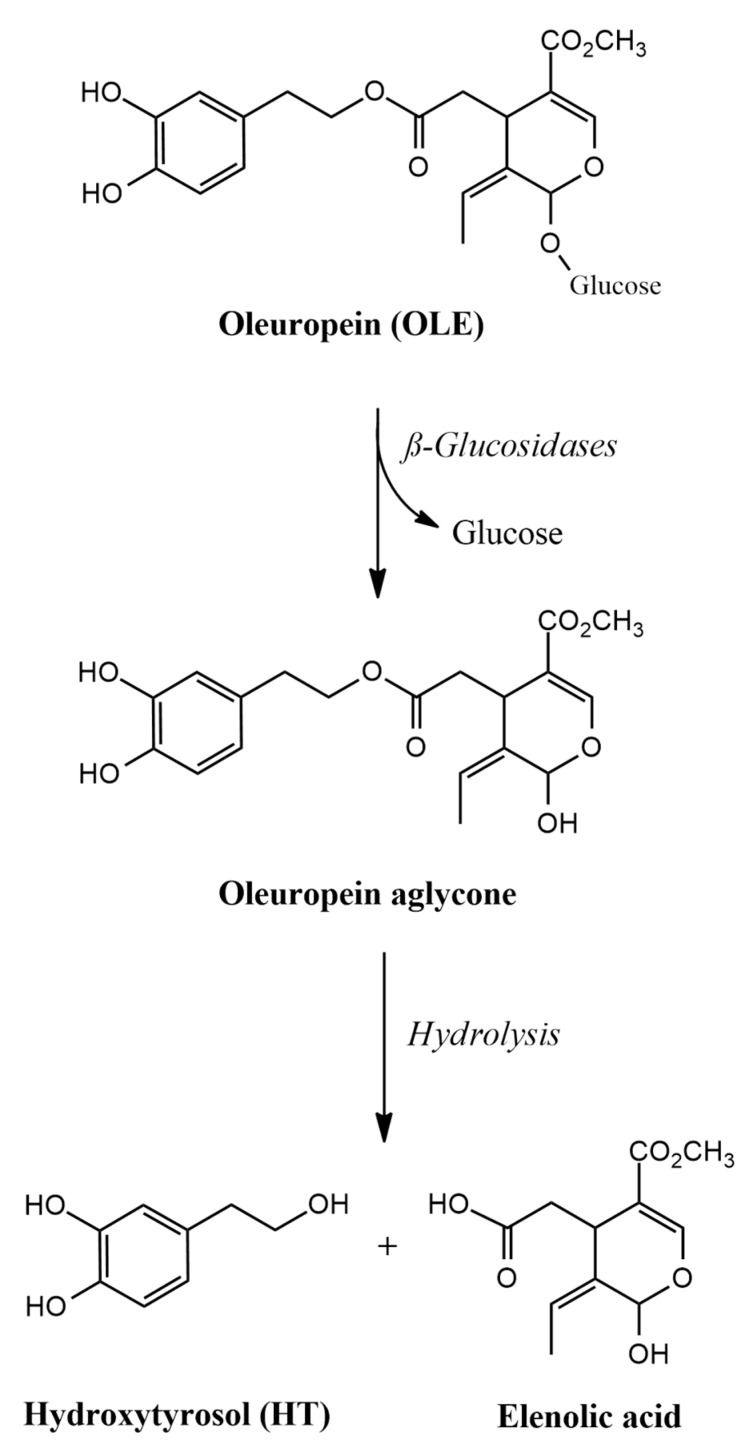
The enzymatic hydrolysis of oleuropein (OLE) produces hydroxytyrosol (HT).

Obviously, olive oil primarily consists of triacylglycerols and approximately 0.5–1.0% non-glycerol constituents, including over 30 phenolic compounds [10]. Similarly to many other plants and edible oils made thereof, the phenolic composition of olive oil is influenced by the production and storage methods, with a higher total phenolic content in extra virgin olive oil compared to refined virgin olive oil. The three predominant phenolic compounds in olive oil are oleuropein (OLE), hydroxytyrosol (HT), and tyrosol, with HT being the major component [11]. When olives mature, the concentration of OLE decreases, while that of HT increases due to the hydrolysis of OLE (Figure 1). The concentrations of HT and OLE in olive oil can vary, with reported ranges of 1.4–5.6 mg/kg and 2.3–9.0 mg/kg, respectively [10]. Other phenolic compounds from olive oil and olive products include luteolin 7-O-glucoside, apigenin 7-O-glucoside, rutin, tyrosol, and verbascoside [12]. Verbascoside is the main hydroxycinnamic derivative in olives, and it increases during the fruit’s maturation [13].

Besides being consumed in the form of olive oils, there are also a variety of other olive-based products. Table olives, for instance, are a popular snack and culinary ingredient available in numerous variations, such as green, black, Kalamata, and stuffed olives filled with ingredients like peppers, garlic, or almonds [14]. Health supplements derived from olives are also popular. Olive leaf extract and olive oil capsules are taken for their potential health benefits, including anti-inflammatory and antioxidant effects [15]. In addition to their culinary uses, olives and olive oil play a significant role in the cosmetic industry. Olive oil is a key ingredient in skincare and haircare products due to its moisturizing and antioxidant properties. It is applied in soaps, lotions, and shampoos, where it nourishes and hydrates the skin and hair [16].

Nutritional studies with human subjects, along with animal and in vitro ones, demonstrated that olive oil-derived OLE and HT exhibit different health-promoting properties [17]. For example, in mice, HT supplementation improved obesity and insulin resistance via the JNK/IRS (Ser 307) pathway, also improving the gut microbiota and enhancing intestinal wall integrity [18]. Moreover, HT supplementation reduced the lipid deposits in liver and muscle by inhibiting the SREBP-1c/FAS pathway, improved oxidative stress by increasing antioxidant enzyme activity, normalized mitochondrial function, and inhibited apoptosis. In diabetic (db/db) mice, HT lowered the fasting glucose levels and serum lipids more effectively than metformin and reduced oxidative damage in liver and muscle tissues [19]. In humans, HT consumption appears to improve lipid profiles and changes in body composition [20,21], while high urinary concentrations of homovanillyl alcohol, an HT metabolite, were linked to a 66% reduced risk of major cardiovascular events and a 19% lower all-cause mortality [22]. Similarly, in animals, OLE was found to attenuate heart failure progression by preventing reductions in cardiac function and antioxidant enzyme levels in rats with induced myocardial infarction [23]. OLE provided cardioprotection against ischemic–reperfusion injuries by significantly reducing the infarct size and improving cardiac function when administered 1 to 3 h before the ischemic event [24]. Moreover, in humans, the intake of OLE was associated with a significantly smaller increase in the blood glucose levels in patients with type 2 diabetes mellitus, also significantly affecting GLP1 and DPP-4 activity [25]. In colonic biopsies from patients with active ulcerative colitis, treatment with OLE significantly reduced the expression of the inflammatory markers COX-2 and IL-17, as well as the IL-17 levels in culture supernatants, suggesting anti-inflammatory effects in the colonic mucosa of ulcerative colitis patients [26].

Moreover, OLE and HT positively affected other health outcomes, such as the nervous and musculoskeletal systems, by mitigating various aspects of aging, including chronic inflammation, decreased autophagy, disrupted protein balance, mitochondrial dysfunction, neural stem cell depletion, and oxidative stress [27]. Additionally, the compounds mentioned demonstrated hepatoprotective effects by reducing elevated liver enzymes, enhancing the antioxidant status of the organism, and attenuating liver inflammation and apoptosis [28,29]. However, both OLE and HT have relatively low bioavailability, and the doses used in animal studies are often relatively high compared to what is administered and/or consumed in human trials [30].

Only a few systematic reviews have summarized the effects of OLE and HT. A review of 12 animal studies highlighted the cardioprotective effects of OLE and olive leaf extract. The included studies demonstrated that OLE and olive leaf extract improved the outcomes in conditions such as hypertension, heart failure, myocardial infarction, renal hypertension, and diabetes. The review suggested a positive effect of OLE and olive leaf extract on cardiovascular health, particularly in animal models [31]. Contrarily, Pastor et al. conducted a meta-analysis to evaluate the global effect of HT on 22 factors related to metabolic syndrome in humans. The findings indicated that HT had a very small effect on evaluated outcomes, and the overall result of the meta-analysis did not support significant conclusions about the intervention’s effectiveness [20]. 

The disparity in the findings between animal studies and human trials highlights the complexity of translating preclinical results to clinical outcomes. While animal studies have consistently shown cardioprotective benefits of OLE and HT, these effects are not as clearly observed in human studies. Furthermore, many human studies, including those reviewed by Pastor et al. [20], often co-administer HT with other bioactive compounds (mainly punicalagin), making it difficult to isolate their specific effects. This lack of control over confounding variables and the relatively small effects observed in human studies contribute to the uncertainty about the precise mechanisms of action of OLE and HT.

Consequently, there seems to still be a gap in the literature and knowledge on the healthiness of olives and especially the frequently advertised compounds OLE and HT. Considering this current gap, although there is substantial existing evidence from various in vitro and in vivo studies involving animals and humans regarding the systemic health-promoting effects of OLE and HT, this systematic review aimed at assessing the available RCT data on the administration of these compounds, whether through standardized or pure-form supplements or enriched foods. The focus was on determining OLE’s and HT’s impact on the overall health outcomes in subjects, healthy or otherwise, who were enrolled in an RCT lasting at least two weeks, including a matched control intervention.

## 2. Materials and Methods

The systematic review followed the PRISMA 2020 guidelines, as outlined by Page et al. [32]. Additionally, it was registered in the International Prospective Register of Systematic Reviews (PROSPERO: CRD42024546850) before starting.

### 2.1. Literature Search

The prominent databases Embase, Scopus, PubMed, and Web of Science were used to search for relevant articles published until 19 April 2024. The following search query “((oleuropein OR hydroxytyrosol) AND (random* OR rct OR trial)) NOT (review OR mice OR rat)” was used. Two researchers independently conducted all the phases of the systematic review process. Initially, the titles and abstracts of retrieved articles underwent eligibility screening. The full texts of relevant studies were then assessed for inclusion, with any disagreements between the researchers resolved through discussion and consensus. Language restrictions were not imposed. Furthermore, the reference lists of eligible RCTs were screened to identify additional relevant studies.

### 2.2. Eligibility Criteria

The comprehensive Population, Intervention, Comparison, Outcomes, and Study (PICOS) selection criteria used to select relevant studies are presented in Table 1. Studies were excluded when they (i) did not have a control group; (ii) did not include an appropriate placebo/control that resembled the intervention but lacked the presence of OLE and/or HT; (iii) used a non-standardized supplement or non-enriched food; (iv) were conducted in individuals younger than 18 years old or included pregnant women; and (v) were not randomized and/or had a study duration of less than two weeks. Moreover, the systematic review did not include trial protocols, observational studies, case reports, case series, in vitro studies, animal experiments, or abstracts without findings.

### 2.3. Data Extraction

Initially, two authors independently screened the titles and abstracts of the studies retrieved from the selected online databases. Later, all the relevant data were extracted and corroborated by other authors. The extracted information included, but was not limited to, the (1) publication details, such as the first author’s name, publication year, country, and study title; (2) study characteristics, including the study design, number and description of arms, durations of washout and treatment, participant numbers for the intervention and placebo groups, participants completing the study, amount of OLE and/or HT supplemented and the form of supplementation; (3) sample characteristics, encompassing the numbers of male and female participants, mean and range of ages, health status, menopausal status, smoking habits, medication usage, baseline characteristics, methods of dietary assessment, baseline and during-study diets, and levels of physical activity; (4) details regarding the reported outcomes, including the sample type (fasted or post-prandial), pre- and post-intervention values or changes (central measure, dispersion measure, and available *p* values); and (5) the results regarding the outcomes of interest.

### 2.4. Quality Assessment

The methodological quality of the chosen studies was evaluated using the modified Jadad score, a widely adopted tool in research for appraising the methodological robustness of RCTs [33,34]. Scores were assigned to each component: randomization, allocation concealment, double-blinding, and withdrawals and dropouts. The scoring criteria are summarized in Table 2.

## 3. Results

### 3.1. Literature Search and Data Extraction

**Figure 2:**
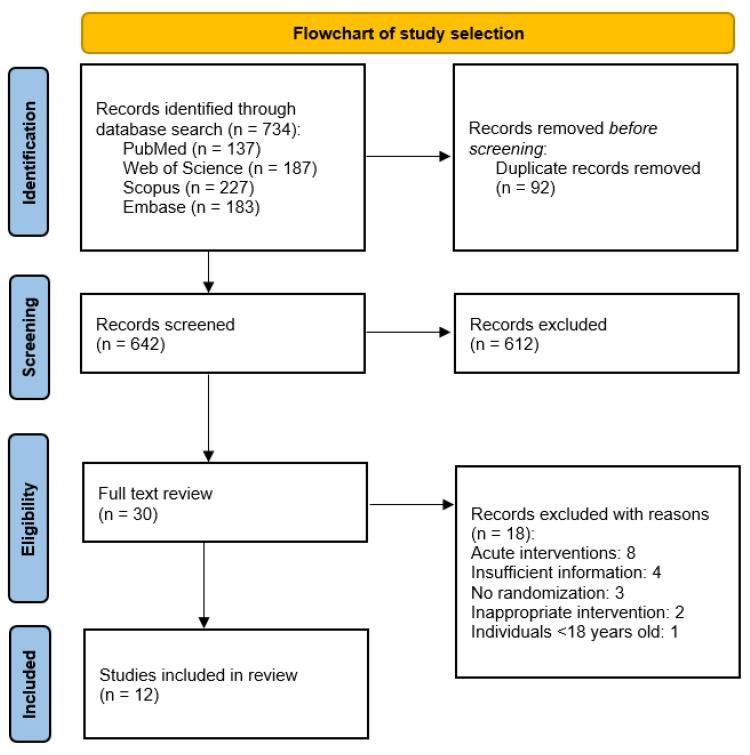
PRISMA flowchart illustrating the selection process for relevant studies in this systematic review.

Figure 2 illustrates the process for identifying relevant studies. Initially, 734 studies were identified based on the pre-determined search criteria. After removing duplicate articles (92 studies), 642 studies were screened based on their titles and abstracts. Of these, 612 studies were excluded due to their focus on pharmacokinetics and bioavailability, in vitro and in vivo animal studies, lack of relevance to the systematic review’s objective, or being literature reviews. Consequently, 30 studies underwent eligibility screening. Among them, eight studies were excluded for evaluating an acute intervention, four studies for providing insufficient information about the intervention, three studies for not being randomized, two studies for evaluating inappropriate interventions (such as (poly)phenol-enriched extra virgin olive oil non-standardized in either OLE or HT, or olive pomace-enriched products non-standardized in either OLE or HT), and one study for including individuals under 18 years old. Ultimately, 12 studies met the eligibility criteria and were included in this systematic review for analysis.

Table 3 presents an overview of the studies included in this systematic review. Six studies evaluated the impact of supplementation with HT [35,36,37,38,39,40], three studies assessed the impact of supplementation with OLE [41,42,43], while three studies evaluated the effect of supplementation with both compounds [44,45,46]. Eight studies included supplements (standardized in OLE/HT or pure compounds) [36,37,38,41,42,43,44,45], while four included enriched foods (desert olive tree pearls, sunflower oil, olive leaf infusion, and bread) [35,39,40,46]. The quantity of the consumed enriched foods ranged from 6 g/d desert olive tree pearls (97.2 md/d HT) [40] to 990 mL/d enriched infusion (320.8 mg/d OLE and 11.9 mg/d HT) [46]; the quantity of ingested standardize supplements ranged from 250 mg/d of either olive extract (100 mg OLE) [41] or olive leaf extract (100 mg OLE) [43] to 1,650 md/d olive oil with 10 mg/d HT [38]; lastly, the quantity of ingested pure compounds ranged from 5 mg/d HT [37] to 15 mg/d HT [36,37]. The supplementation period with a standardized supplement, a pure compound or enriched foods ranged from three weeks in two studies [35,36] to 12 months in one study [41]. 

Among the selected clinical trials, six studies employed a crossover group design [35,36,38,44,45,46], while the other six studies employed a parallel group design [37,39,40,41,42,43]. One study was unblinded [46], two were single-blinded [35,39], and nine studies were double-blinded [36,37,38,40,41,42,43,44,45]. The studies included in this systematic review had 683 participants, with 348 in the intervention groups and 335 in the control groups, focusing on different health conditions and demographics. The participants ranged from healthy individuals [35,36,40] to those with specific health conditions, such as overweight or obesity [37,39,42,44], prediabetes [46], postmenopausal osteopenia [41], pre-hypertension [45], chronic coronary artery syndrome [38], and knee pain [43].

### 3.2. Outcomes

This systematic review included a range of studies that examined diverse health outcomes of OLE and HT from either supplements or enriched foods among various demographics (Table 3). These studies were grouped according to their primary focus on health indicators such as CVD risk factors, liver parameters, and bone, joint, and cognitive health. Ten studies focused on assessing CVD risk factors in diverse populations [35,36,37,38,39,41,42,44,45,46], and three studies focused on assessing liver function [39,42,44]. In contrast, one study each focused on bone health [41], joint functionality [43], and cognitive health [40]. Vázquez-Velasco et al. [35] evaluated the effects of 10–15 g/d of HT-enriched sunflower oil (45–50 mg HT) on healthy subjects’ lipid profiles and inflammatory markers. de Bock et al. [44] investigated the impact of 51.1 mg/d OLE and 9.7 mg/d HT on insulin sensitivity, lipid profile, hormones, adiposity, blood pressure, and liver parameters in overweight individuals. Colica et al. [36] investigated the antioxidant effects and metabolic state associated with 15 mg/d HT administration in healthy participants. Lockyer et al. [45] studied pre-hypertensive subjects to examine the effects of 136.2 mg/d OLE and 6.4 mg/d HT on blood pressure, lipid profile, glucose metabolism, and inflammatory markers. Araki et al. [46] included prediabetic subjects to assess the impact of 990 mL/d olive leaf infusion (320.8 mg OLE and 11.9 mg HT) on anthropometric parameters, glucose metabolism, and lipid profile changes. Stevens et al. [42] evaluated the effects of 500 mg/d olive leaf extract (83.5 mg OLE) on the anthropometric parameters, lipid profile, blood pressure, glucose metabolism, and liver parameters in overweight or obese individuals. Fytili et al. [37] focused on the effects of 5 or 15 mg/d HT on anthropometric parameters in overweight or obese subjects. Binou et al. [39] assessed the impact of 60 g/d HT-enriched whole wheat bread (32.5 mg HT) on CVD risk factors and metabolic parameters in overweight or obese subjects with type 2 diabetes mellitus (T2DM). Ikonomidis et al. [38] studied individuals with chronic coronary artery syndrome to evaluate the effects of 1,650 mg olive oil and 10 mg/d HT on vascular function, oxidative stress, inflammatory biomarkers, and lipid profile. Filip et al. [41] investigated the effects of 250 mg/d olive extract (100 mg OLE) and 1000 mg Ca on lipid profile and serum inflammatory markers in postmenopausal and osteopenic women, also focusing on bone metabolism. Finally, Horcajada et al. [43] evaluated the effects of 250 mg/d olive leaf extract (100 mg OLE) on the joint functional capacity, cartilage degradation, and inflammation in subjects with knee pain, aiming to understand its potential benefits for joint health. At the same time, Yoon et al. [40] examined the impact of 6 g/d desert HT-enriched olive tree pearls (97.2 mg HT) on cognitive function in healthy middle-aged and older individuals, using the Cognitrax test to assess multiple cognitive domains.

### 3.3. Risk of Bias Assessment

The results of the methodological quality assessment using the modified Jadad score are shown in Table 4. One study was categorized as low quality, with a score of 2 [46]; five studies were categorized as medium quality, with a score of 4–5 [35,36,39,41,43]; whereas six studies were categorized as high quality, with a score of 6–7 [37,38,40,42,44,45]. Four studies received the highest score of 7, indicating that all the criteria had been met [38,42,44,45]. The average score for the studies was 5.54 (SD = 1.57). Therefore, considering the mean score, the quality of the included studies was assessed as moderate.

## 4. Discussion

### 4.1. Cardiovascular Disease Risk Factors

#### 4.1.1. Effects of OLE and HT Supplementation on Anthropometric Parameters

Body weight is a risk factor for CVD. Dong et al. showed that obesity and significant weight changes (weight gain > 4%) were associated with higher risks of CVD in patients with hypertension [47]. A meta-analysis revealed that olive oil consumption does not significantly affect the overall body fat distribution. However, capsule supplementation showed a slight increase in adipose mass and waist circumference, with a decrease in auxiliary culinary use. Lean mass was negatively impacted by higher doses and longer durations of olive oil intake [48]. The studies with regard to pure OLE and HT supplementation revealed varying effects on body composition. Colica et al. found that a daily intake of HT for 3 weeks significantly reduced the weight, fat mass percentage, and suprailiac skinfold in healthy subjects [36]. Fytili et al. demonstrated that a 6-month supplementation of HT led to significant reductions in body weight, body fat, and visceral fat mass in overweight/obese subjects compared to the control group [37]. Binou et al. showed that HT-enriched whole wheat bread for 12 weeks significantly reduced the body weight, body fat, and waist circumference of overweight/obese and T2DM subjects. The intervention group experienced greater reductions, especially in body fat mass, which reached statistical significance. No changes in lean body mass were observed [39]. The positive results reported in the studies can be attributed to several factors: participants received regular consultations with a dietitian, ensuring adherence and personalized guidance; they followed a hypocaloric diet tailored to their basal metabolic rate, which naturally promoted weight loss; and they adhered to the principles of the Mediterranean diet, known for its health benefits, which likely complemented the effects of OLE and HT supplementation.

However, studies in rat models have revealed either the prevention of obesity (and increase of fat mass) or a decrease in visceral fat levels in obese mice after HT supplementation. Peyrol et al. highlighted that HT could improve lipid profiles, glycaemia, and insulin sensitivity, which are crucial in managing body weight and obesity-related issues. HT targeted multiple molecular pathways to exert these benefits despite its low bioavailability [49]. Wang et al. demonstrated that HT supplementation prevented fine particular matter (PM_2.5_)-induced adiposity and insulin resistance in mice. HT inhibited visceral fat accumulation, oxidative stress, hepatic inflammation, and NF-κB activation. It also enhanced the gut microbiota, contributing to its beneficial effects on adiposity and metabolism [50]. Fki et al. found that HT-rich extracts from olive leaves reduced the body weight and adipose tissue mass in rats on a high-fat diet. These extracts also improved the lipid profile, lowered the liver enzyme levels, enhanced the antioxidant status, and reduced liver inflammation and apoptosis, suggesting hypolipidemic and hepatoprotective effects against diet-induced metabolic disorders [28]. In vitro models have demonstrated that HT helps in downregulating genes related to adipogenesis, thus protecting adipocytes from excessive growth and enlargement. Consuming HT enhances the oxidative status of adipocytes and increases their metabolism by promoting mitochondrial biogenesis [51].

HT exerts its antioxidant effects through several interconnected mechanisms. It enhances the body’s antioxidant defenses by stimulating the expression and activity of crucial antioxidant enzymes such as catalase (CAT), superoxide dismutase (SOD), and glutathione peroxidase (GPx) [52]. HT achieves this by regulating the gene expression of these enzymes and activating nuclear factor erythroid 2 (Nrf2), a key transcription factor that drives the production of various phase II detoxifying enzymes [53]. Additionally, HT improves mitochondrial function and promotes mitochondrial biogenesis by activating peroxisome proliferator-activated receptor coactivator 1α (PPARGC1α). This activation enhances the energy production and overall function of mitochondria, helping to counteract oxidative damage [54].

In contrast to the positive results presented, de Bock et al. found that supplementation with OLE and HT in healthy subjects for 3 weeks resulted in no changes in body composition [44]. Araki et al. reported that consuming olive leaf infusion rich in OLE and HT had no significant impact on the body weight and waist circumference in prediabetic subjects [46]. Similarly, Stevens et al. observed no significant changes in the body weight, BMI, or waist-to-hip ratio after 8 weeks of OLE-standardized olive leaf extract in overweight/obese individuals [42]. In these studies, participants were asked to follow their normal diet and did not receive consultation from a dietitian, contrasting with the positive results mentioned earlier. The lack of dietary guidance and personalized nutritional support likely contributed to the absence of significant findings. Unlike the studies where participants were supported by dietitian consultations and followed a hypocaloric Mediterranean diet, these studies did not involve any structured dietary intervention. This suggests that the success of OLE and HT supplementation may be significantly enhanced when combined with professional dietary advice and a controlled eating plan, emphasizing the importance of a holistic approach to weight management and health improvement. 

However, animal data support the positive effects of OLE supplementation on anthropometric parameters. For example, der Stelt et al. investigated the impact of oleuropein supplementation in mice fed a high-fat diet [55]. They found that oleuropein prevented body weight gain, resulting in weights comparable to those of mice on a regular-fat diet. The benefits appeared to result from an initial decrease in intestinal energy uptake and a subsequent increase in satiety signals, indicating a reduction in adiposity. Mikami et al. studied the effects of olive leaf extract rich in OLE in physically inactive mice on a high-fat diet. They found that the extract suppressed increases in fat mass and body weight. These benefits are likely due to the improvement of mitochondrial function by oleanolic acid and the antioxidant capacity of oleuropein from olive leaf extract [56]. Nevertheless, it should be mentioned that a high dose of oleuropein was used in animal studies, equivalent to a human dose of 61.5 mg/kg or 3.1 g of OLE consumption daily [55]. In contrast, the one used in human studies was 50–136 mg OLE/d (Table 2).

#### 4.1.2. Effects of OLE and HT Supplementation on Lipid Levels

LDL-C is an important causal risk factor for atherosclerotic CVD [57]. A systematic review and dose–response meta-analysis of randomized trials found that increasing olive oil consumption by 10 g/day had minimal effects on blood lipids, including TC, LDL-C, HDL-C, and TAG. The TC levels showed a slight increase with up to 30 g/day of olive oil but plateaued afterward. HDL-C showed a minor non-linear increase, peaking at 20 g/day. Overall, olive oil consumption had minimal impact on the serum lipid levels in adults, and further large-scale randomized trials are needed for more reliable results [58]. 

The studies on supplementation with various forms of OLE and HT presented mixed results regarding their effects on lipid profiles. Filip et al. observed significant benefits in postmenopausal and osteopenic women who took OLE-standardized olive extract and calcium for 12 months [41]. This group showed significant reductions in TC and LDL-C compared to a control group, although the TAG levels increased in the control group and slightly decreased in the treatment group. Lockyer et al. demonstrated that a 6-week OLE and HT intake period significantly reduced the plasma TC, LDL-C, and TAG from baseline in pre-hypertensive subjects. Still, the HDL-C levels decreased without significant effects compared to the control [45]. Araki et al. reported that drinking olive leaf infusion rich in OLE and HT for 12 weeks significantly reduced the LDL-C levels in prediabetic subjects, with no changes in TAG and HDL-C compared to a control group [46]. Ikonomidis et al. found that a 4-week supplementation with olive oil and HT in subjects with chronic coronary artery syndrome significantly decreased the TAG levels compared to baseline but did not affect the lipoprotein(a), LDL-C, HDL-C, and TC levels [38]. Finally, Binou et al. observed significant decreases in the TC and LDL-C levels in overweight, obese, and T2DM subjects who consumed HT-enriched whole wheat bread for 12 weeks. However, no other significant changes or differences were found [39]. In contrast, Vázquez-Velasco et al. found that supplementing with HT-enriched sunflower oil for 3 weeks did not alter the serum TC and lipoprotein levels in healthy subjects [35]. Similarly, de Bock et al. reported that a daily intake of OLE and HT over 12 weeks did not improve the lipid profiles in overweight subjects [44]. Colica et al. found no significant changes in the TAG, TC, and HDL-C levels in healthy subjects after supplementing with HT for 3 weeks. However, a positive correlation between TC and LDL-C was noted [36]. Stevens et al. found that supplementing overweight and obese subjects with OLE-standardized olive leaf extract for 8 weeks did not reduce their blood lipid profiles compared to placebo [42]. 

The contrasting results in studies examining the effects of OLE and HT supplementation on lipid profiles can be attributed to several key factors. Firstly, the dosage of OLE and HT varied across the studies, which likely influenced the outcomes. Higher doses may be necessary to elicit significant changes in lipid profiles, while lower doses might not be sufficient to produce noticeable effects. The European Food Safety Authority (EFSA) has concluded that individuals should consume at least 5 mg of HT and its derivatives in olive oil daily to reduce LDL-C levels [59]. Secondly, the type of intervention, whether through supplements or enriched foods, plays a role. Enriched foods might have different absorption rates and interactions with other dietary components compared to supplements, affecting their efficacy. In this sense, Bender et al. showed that dietary HT administered through the food supplements is more bioavailable and its bioavailability increases with the administered dose [30]. de Bock et al. found that OLE resulted in higher plasma concentrations when taken in liquid form compared to capsules. Although there was no significant difference in the peak HT concentrations between the liquid and capsule forms, the peak concentration was reached earlier with the liquid preparation [60]. Nonetheless, López de las Hazas et al. showed that OLE may be the most appropriate precursor of HT for inclusion in foods or nutraceutical products [61]. Thirdly, when considering the composition of olive leaf extract standardized in OLE, it is crucial to acknowledge the presence of other bioactive compounds that can influence the overall effectiveness and health benefits of the extract. The concentration of key phenolic compounds from olive leaves, such as luteolin 7-*O*-glucoside, apigenin 7-*O*-glucoside, rutin, tyrosol, and verbascoside, can vary depending on the extraction method and conditions, potentially leading to variations in the efficacy of OLE products. For example, verbascoside demonstrated antioxidant and anti-inflammatory properties [62,63], also exerting antidepressant effects through various pharmacological mechanisms [64].

Thus, it appears that supplements are generally the most effective way to administer OLE and HT, while enriched foods should preferably use OLE to deliver the intended benefits. Moreover, OLE and HT have low populational dietary intake, poor bioavailability, and high inter-individual variability after absorption through the gastrointestinal tract, which could restrict the full benefits of these compounds [30]. Lastly, the baseline characteristics of participants also influenced the results. Differences in health status, age, and baseline lipid levels mean that individuals with pre-existing conditions, such as hyperlipidemia or metabolic syndrome, might respond differently to supplementation compared to healthy individuals. Not least, other confounding factors, such as variations in participants’ diets, lifestyles, and adherence to intervention protocols, also contributed to the mixed outcomes.

Regarding the available data from the scientific literature on olive oil and its phenolic compounds, the prospective EUROLIVE study demonstrated that daily administration of 25 mL of olive oil with varying phenolic content (low 2.7 mg/kg, medium 164 mg/kg, and high 366 mg/kg) decreased LDL-C and TAG, and increased HDL-C, in a dose-dependent manner. The study’s results suggested that the phenolic content of olive oil provides additional benefits for plasma lipid levels and oxidative damage beyond those offered by monounsaturated fats alone [65]. On the one hand, in animal studies, Jemai et al. observed that HT and its derivative, triacetylated-HT, effectively lowered the cholesterol levels and reduced lipid peroxidation in Wistar rats [66]. Meanwhile, Tabernero et al. found that HT, HT-acetate, and ethyl-HT ether improved the lipid profiles, reduced oxidative stress, and modulated inflammatory markers in rats fed a cholesterol-rich diet, with HT-acetate showing the most promising effects [67]. HT could potentially lower the levels of SREBP-1c, a key controller of fatty acid and cholesterol production in the liver [19]. 

On the other hand, also considering data from animal studies, in a study by Park et al., mice fed a high-fat diet supplemented with OLE exhibited reduced plasma and hepatic lipid levels compared to those on a high-fat diet alone. The expression of genes involved in lipid metabolism and inflammation was also altered favorably in the liver of oleuropein-supplemented mice [68]. Another study by Olmez et al. demonstrated that rats fed a cholesterol-enriched diet experienced elevated cholesterol levels, which were significantly reduced by supplementation with olive leaf extract, a rich source of OLE. Olive leaf extract influenced liver mRNA expression of acyl-CoA oxidase along with PPARα suggesting that it could enhance fatty acid oxidation and improve overall lipid metabolism in the liver, contributing to better metabolic health [69]. These findings suggest that OLE may protect against hepatic steatosis and atherosclerosis by modulating lipid metabolism and reducing cholesterol levels.

#### 4.1.3. Effects of OLE and HT Supplementation on Glycemic Parameters

Elevated blood glucose levels, even within the normal to borderline range, were linked to a higher risk of CVD and mortality in adults who had not been diagnosed with diabetes [70]. A human intervention study showed that consumption of olive oil rich in phenols could improve post-prandial blood glucose levels [71]; however, a systematic review and meta-analysis found that olive oil intake had no significant effect on FBG, insulin levels, or HOMA-IR. While a decreasing trend was noted in these outcomes, subgroup analyses by age, health status, dose, and duration of extra virgin olive oil intake did not significantly alter the results [72].

The studies on supplementation with various forms of OLE and HT showed mixed results. de Bock et al. found that supplementation with OLE and HT in healthy subjects for 3 weeks resulted in a 15% improvement in insulin sensitivity and a 28% improvement in pancreatic β-cell function [44]. Additionally, there was a reduction in the area under the curve for both glucose and insulin. Araki et al. noted a significant decrease in the fasting blood glucose levels in prediabetic subjects after 12 weeks of supplementation with an olive leaf infusion, rich in OLE and HT, but no substantial changes in other glycemic markers [46]. Binou et al. observed significant decreases in glucose and HbA1c levels in overweight/obese and T2DM subjects after 12 weeks of supplementation with HT-enriched whole wheat bread, with a significantly greater decrease in the fasting glucose levels in the intervention group compared to the control group, and a marginally significant difference in HbA1c levels between the intervention and control groups [39]. However, Colica et al. observed no significant changes in healthy subjects after supplementation with HT for 3 weeks [36]. Lockyer et al. reported no effects on the fasting glucose, insulin, or related indices in pre-hypertensive subjects after 6 weeks of supplementation with OLE and HT [45]. Stevens et al. found no significant difference in changes in the fasting blood glucose and insulin levels between intervention and control groups of overweight/obese subjects after 8 weeks of supplementation with OLE-standardized olive leaf extract [42]. 

OLE and HT demonstrated antidiabetic effects in various studies. Jemai et al. found these compounds reduced the serum glucose and cholesterol levels while restoring the antioxidant balance in diabetic rats [73]. HT can enhance glucose tolerance and increase insulin sensitivity, leading to a decrease in HOMA-IR [74]. López-Villodres et al. observed that HT lowered oxidative and nitrosative stress, reduced inflammation, and influenced biochemical processes associated with diabetic vasculopathy in streptozotocin-induced diabetic rats [75]. Qadir et al. investigated the effects of pure oleuropein on alloxan-induced type 1 diabetic rats. They found that it significantly attenuated the blood glucose levels and enhanced the in vivo antioxidants, increasing the serum glutathione levels, which is critical in protecting β cells from oxidative damage caused by free radicals [76]. Also, OLE protected β cells against induced damage and promoted their regeneration, possibly by scavenging oxidative stress and reactivating insulin secretion. Additionally, oleuropein indirectly enhances antioxidant defenses by stimulating the expression of intracellular antioxidant enzymes through the activation of the Nrf2 pathway. It also increases the levels of non-enzymatic antioxidants like glutathione, α-tocopherol, β-carotene, and ascorbic acid, further increasing its antioxidant capacity [77]. These findings suggest OLE’s potential as a hypoglycemic and antioxidant agent, offering protection and functional improvement of β cells in diabetic conditions.

The contrasting outcomes reported in this systematic review may be caused by differences in the baseline values within the studied population, as the glucose and insulin levels were all within normal ranges and the study design did not include a glucose challenge test [42]. Also, in studies where participants followed a hypocaloric diet, improvements in glucose metabolism may be due to the diet rather than the supplements. The results vary depending on the health status of participants, with the benefits more apparent in those with metabolic disturbances like prediabetes or T2DM. Furthermore, the lack of significant effects observed could be attributed to the particular mechanism of action of OLE. In addition to inhibiting α-glucosidase activity [78], OLE also blocks intestinal glucose receptors, resulting in decreased glucose absorption. However, when supplemented after meals, OLE may compete with glucose released from food in the gastrointestinal tract for glucose receptors, thereby partially decreasing its absorption and mechanism of action [79].

#### 4.1.4. Effects of OLE and HT Supplementation on Blood Pressure

Among the risk factors for CVD, high blood pressure has the most substantial evidence of causing these diseases and is a prevalent risk factor [80]. A systematic review on the effects of olive oil on blood pressure in individuals without previous cardiovascular events found that liquid olive oil, particularly extra virgin olive oil at doses between 10 mL and 50 mL per day, significantly reduced diastolic blood pressure by −0.73 mm Hg, with no statistically significant decrease in systolic blood pressure [81]. Contrarily, a more recent meta-analysis found out that the consumption of extra virgin olive oil had no significant effect on blood pressure [82].

Among the included studies, Lockyer et al. reported that supplementation with OLE and HT in pre-hypertensive subjects for 6 weeks led to significant reductions in the 24 h SBP and DBP, and in the daytime SBP and DBP, compared to control, with no significant differences between the two treatments in terms of the nighttime BP [45]. However, Stevens et al. observed no significant changes in the blood pressure parameters between intervention and control groups of overweight/obese subjects after 8 weeks of supplementation with OLE-standardized olive leaf extract [42]. Moreover, de Bock et al. found that supplementation with OLE and HT in overweight subjects for 12 weeks resulted in no significant changes in ambulatory blood pressure [44]. Lastly, even though Binou et al. reported significant decreases in the SBP and DBP in overweight/obese and T2DM subjects after 12 weeks of supplementation with HT-enriched whole wheat bread, there was no statistical significance in these parameters between the two groups because both groups were trained in essential parameters of the study protocol, such as meal planning, portion sizes, and counselling on the principles of the MD [39].

The presented contrasting results might have multiple reasons. Firstly, the positive results from Lockyer et al. should be interpreted with caution because data for 15 subjects were missing, which could skew the results and potentially overestimate the effectiveness of the supplementation. Secondly, the lack of significant effects in the studies by Stevens et al. and de Bock et al. might be attributed to the participants’ baseline BP levels being around 130/80 mmHg. This level of blood pressure might not allow for significant improvement, or the supplementation may have been insufficient to produce a notable effect in these individuals. Lastly, the duration and dosage of the supplementation varied across studies, contributing to the differing outcomes. Shorter intervention periods or lower dosages might not be enough to elicit significant changes in BP.

Regarding the data from the wider literature, Hermans et al. assessed the impact of a two-month supplementation of Tensiofytol^®^ (100 mg/d OLE) on hypertension. The results showed significant reductions in the SBP and DBP. However, the study was an observational, non-controlled, non-randomized pilot study [83]. Romero et al. evaluated the chronic effects of OLE-enriched olive leaf extract on spontaneously hypertensive rats. The OLE treatment reduced the SBP, heart rate, and cardiac and renal hypertrophy. The authors concluded that OLE exerted antihypertensive effects by improving vascular function and reducing oxidative and inflammatory status [84]. Indeed, other previous studies have also demonstrated a decrease in SBP and DBP after consuming OLE [85]; however, those studies typically involved participants with elevated BP levels. In the current systematic review, Lockyer et al. focused on pre-hypertensive subjects [45], while de Bock et al. [44] and Stevens et al. [42] included participants with normal BP. In the study conducted by Binou et al. [39], although pre-hypertensive subjects were included, the amount of HT for a clinically relevant BP outcome might have been too low (32.5 mg/d). Lopez-Villodres et al. found that HT supplementation (10 mg/kg/day for 2 months) increased the nitrite and nitrate levels in diabetic rats, potent nitric oxide donors acting as vasorelaxants [75]. 

#### 4.1.5. Effects of OLE and HT Supplementation on Inflammatory and Oxidative Markers

Inflammatory and oxidative processes are well established as key factors in the development and complications of CVD. High levels of inflammatory and oxidative markers have been shown to predict future cardiovascular events [86]. In a meta-analysis, no significant effect of consuming extra virgin olive oil on the C-reactive protein, interleukin-6, interleukin-10, and tumor necrosis factor α levels was noticed [82].

In the context on OLE and HT supplementation, Vázquez-Velasco et al. found that supplementation with HT-enriched sunflower oil in healthy subjects for 3 weeks significantly decreased the sVCAM-1 levels during the trial period compared to the control period [35]. Ikonomidis et al. noted a decrease in inflammatory markers, including high-sensitivity C-reactive protein (hs-CRP) and IL-6, after supplementation with olive oil and HT in chronic coronary artery syndrome subjects for 4 weeks, with a greater reduction compared to placebo. Also, a significant decrease in markers of oxidative stress was observed. The ox-LDL and MDA levels were significantly reduced after supplementation with olive oil and HT compared to baseline [38]. Binou et al. found a significant decrease in the adiponectin and TNF-α levels in overweight/obese and T2DM subjects after 12 weeks of supplementation with HT-enriched whole wheat bread. However, there were no significant changes in the hs-CRP levels between the intervention and control groups [39]. Lockyer et al. reported that supplementation with OLE and HT in pre-hypertensive subjects for 6 weeks had no effect on various inflammatory markers except for a significant reduction in plasma IL-8 compared to the control [45]. de Bock et al. found that supplementation with OLE and HT in overweight subjects for 12 weeks resulted in a significant decrease in the IL-6 levels between the intervention and control groups, while no changes were observed in the IL-8, hs-CRP, and TNF-α levels between the two groups [44]. Contrarily, Filip et al. observed no significant differences in inflammatory markers, including hs-CRP and IL-6, following a 12-month supplementation of OLE-standardized olive extract and calcium in postmenopausal and osteopenic women [41]. Likewise, Horcajada et al. found no differences in the evaluated inflammatory markers (IL-8, TNF-α, and PGE2) after supplementation with OLE-standardized olive leaf extract in subjects with knee pain for 6 months [43], while Colica et al. found that supplementation with HT in healthy subjects for 3 weeks did not result in significant changes in the ox-LDL and MDA levels [36]. 

The contrasting results highlight the complexity of determining the efficacy of HT and OLE supplementation on inflammatory and oxidative markers. The baseline health status of participants varies significantly across studies. For example, the positive effects observed by Vázquez-Velasco et al. [35] and Ikonomidis et al. [38] were in healthy and chronic coronary artery syndrome subjects, respectively, while the neutral findings by Filip et al. [41] and Horcajada et al. [43] involved postmenopausal, osteopenic women, and individuals with knee pain. This variability can influence the outcomes, as different populations might respond differently to HT and OLE supplementation. Moreover, different studies measured various markers of inflammation and oxidative stress, which might not all be equally sensitive to the effects of HT and OLE. For example, while IL-6 and IL-8 showed some changes in certain studies, others like hs-CRP, TNF-α, and PGE2 did not show consistent changes. Lastly, the study designs, including the control conditions and additional interventions, vary. Binou et al.’s study [39], for example, included dietary counselling that could have influenced the results, making it hard to isolate the effects of HT-enriched bread. All these differences contributed to the variability in outcomes.

Several animal studies indicated that OLE and HT help regulate tissue inflammation and oxidative stress. For example, Giner et al. found that oleuropein improved clinical symptoms, reduced tumor growth, and decreased inflammatory markers (IL-6, IFN-γ, TNF-α, and IL-17A), and proteins associated with cancer pathways. Additionally, oleuropein inhibited the Th17 response in acute colitis, suggesting it could be a protective dietary supplement against colitis-associated colorectal cancer [87]. Huguet-Casquero et al. focused on oleuropein’s efficacy in treating inflammatory bowel diseases using nanostructured lipid carriers (NLCs) to enhance its delivery to the inflamed colon. Their study showed that NLC-loaded oleuropein (NLC-OLE) was more effective than conventional oleuropein in reducing TNF-α secretion, reactive oxygen species in macrophages, and inflammation in a murine model of acute colitis. NLC-OLE also improved the histopathology of the colon, indicating its potential as a targeted drug delivery system for inflammatory bowel disease treatment [88]. 

Moreover, Yu et al. studied HT’s impact on acute liver injury. They demonstrated that HT treatment reduced pro-inflammatory M1 macrophages and increased anti-inflammatory M2 macrophages after lipopolysaccharide stimulation, lowering the levels of inflammatory cytokines (TNF-α, IL-1β, IL-6, IL-10, and IL-4) while enhancing anti-inflammatory ones through suppression of the ERK pathway. In vivo, HT mitigated liver inflammation and improved liver function, suggesting its potential as a treatment for acute liver injury by modulating macrophage-driven inflammation [89]. Lastly, Jin et al. focused on OLE’s protective effects against myocardial ischemia/reperfusion injury in rats. OLE reduced the myocardial infarction size, creatine kinase-MB, and lactate dehydrogenase levels. It also decreased the inflammatory markers (TNF-α, IL-1β, IL-6) and oxidative stress markers (malondialdehyde) while increasing the antioxidant enzymes (SOD, GSH, CAT). Mechanistically, oleuropein inhibited pathways involving p53, p-MEK, p-ERK, and p-IκBα proteins [90]. Together, these findings highlight the promising anti-inflammatory and antioxidant properties of OLE and HT, making them valuable for various therapeutic applications.

### 4.2. Liver Function

Non-alcoholic fatty liver disease (NAFLD), the predominant chronic liver condition, is strongly linked with insulin resistance, obesity, and metabolic syndromes [91]. A systematic review examining the effects of olive oil on liver health revealed that consuming olive oil resulted in notable improvements in hepatic steatosis, evidenced by lower ultrasound grading and reduced levels of aspartate transaminase and alanine transaminase [92].

Regarding OLE and HT supplementation, Binou et al. observed a significant reduction in the AST and ALT levels in overweight/obese and T2DM subjects after 12 weeks of HT-enriched whole wheat bread supplementation, but no change in the GGT levels. However, the changes in AST and ALT between the intervention and control groups were not statistically significant [39]. On the contrary, de Bock et al. showed that 12-week supplementation with OLE and HT in overweight subjects resulted in no differences in the AST, ALT, alkaline phosphatase (ALP), or GGT levels between intervention and control groups [44]. Stevens et al. reported that 8-week supplementation with OLE-standardized olive leaf extract in overweight/obese subjects did not significantly affect the serum ALP, GGT, AST, ALT, and bilirubin levels, which remained within normal ranges compared to the control [42].

Clinical trials involving humans showed conflicting results regarding the effect of olive oil consumption on liver markers. A meta-analysis of RCTs found no significant differences in the AST and ALT levels after olive oil supplementation [93]. However, animal studies suggested that olive oil and its phenols positively affect liver function. For example, Varela-Lopez et al. showed that a lifelong diet of virgin olive oil helps protect the liver as mice age [94]. Fki et al. [28] and Mahmoudi et al. [29] explored the protective effects of OLE- and HT-rich extracts from olive leaves on a high-fat diet and bisphenol A-induced liver injury in rats, respectively. Both studies found that treatment with OLE and HT reduced elevated liver enzymes such as AST and ALT, indicating a protective effect against liver damage. The supplementation also improved the lipid profiles and reduced markers of inflammation and apoptosis in the liver, suggesting a hepatoprotective and hypolipidemic effect.

Moreover, Miao et al. investigated the effect of HT on acute liver injury induced by lipopolysaccharide in mice. They found that HT treatment decreased the levels of ALT and AST, indicating reduced liver injury. HT also modulated macrophage polarization and inhibited the TLR4/NF-κB pathway, demonstrating its anti-inflammatory and antioxidant properties in acute liver injury [95]. Lastly, Santini et al. focused on OLE’s effects on liver damage in the context of NAFLD in mice fed a high-fat diet. They found that OLE reduced the liver weights and improved the inflammation and oxidative stress markers, indicating its potential to ameliorate liver damage associated with NAFLD [96]. However, considering the previously mentioned meta-analysis [93], further research is needed to substantiate the animal results in humans.

### 4.3. Bone and Joint Health

Bone and joint health represent crucial aspects of overall well-being and mobility, particularly as individuals age. Maintaining these elements is essential for preventing discomfort and preserving the ability to perform daily activities. Research indicates that olive oil and its derivatives, known for their anti-inflammatory, antioxidant, and autophagy-enhancing properties, may offer therapeutic potential for osteoarthritis [97]. In this context, Filip et al. found that 12-month supplementation with OLE-standardized olive extract and calcium in postmenopausal and osteopenic women resulted in a significant 32% increase in the osteocalcin (OC) serum levels in the treatment group compared to a 6% decrease in the placebo group. The OC/CTX (C-terminal cross-linking telopeptide of type I collagen) ratio significantly decreased in the placebo group, indicating increased bone-resorption activity, while no significant change was observed in the treatment group. The lumbar spine bone mineral density decreased in both groups, and the femur neck bone mineral density did not change significantly [41]. However, Horcajada et al. reported that 6-month supplementation with OLE-standardized olive leaf extract in subjects with knee pain did not improve the Global Knee Injury and Osteoarthritis Outcome Score (KOOS), KOOS subscores, pain intensity at rest, or during walking substantially. The serum cartilage biomarkers Coll2-1NO2 and Coll2-1 increased, while the CTX and OC levels remained stable, with no significant differences between the treatment and placebo groups [43].

Studies on the impact of olive oil on bone and joint health are limited, as the majority of research in this area focuses mostly on calcium, vitamin D, and omega-3 fatty acids. However, Cardoso et al. examined the effects of extra virgin olive oil and the traditional Brazilian diet on the bone mineral density in severely obese adults, finding beneficial outcomes after a 12-week intervention [98]. Similarly, Hekmatpou et al. investigated the efficacy of olive oil in reducing morning inflammatory pain in women with rheumatoid arthritis, reporting significant improvements in pain, swollen joints, and overall disease activity [99]. 

In addition, few studies have investigated the therapeutic potential of OLE in alleviating symptoms and preventing the progression of bone and joint diseases. For example, Impellizzeri et al. showed OLE’s efficacy in reducing inflammation and tissue damage associated with collagen-induced arthritis in mice. Treatment with OLE ameliorated the clinical signs and improved the histological status in the joints and paws of mice with collagen-induced arthritis, along with reducing the oxidative and nitrosative damage and plasma levels of proinflammatory cytokines [100]. Takuma et al. explored the chondroprotective effects of olive leaf extract rich in OLE, finding that the extract’s supplementation prevented cartilage degeneration in mice with knee osteoarthritis, potentially by modulating hyaluronan metabolism in synovial cells [101]. Lastly, Feng et al. investigated the effects of OLE on human osteoarthritis chondrocytes, revealing its ability to inhibit inflammation and cartilage degradation induced by interleukin-1β by suppressing NF-κB and MAPK signaling pathways [102]. Therefore, these findings collectively suggest the therapeutic potential of OLE in managing bone and joint diseases by targeting inflammatory pathways and protecting cartilage integrity. However, further studies are necessary to substantiate these results.

### 4.4. Cognitive Health

The Mediterranean diet, which includes olive oil, is increasingly recognized for its potential benefits in terms of cognitive health, particularly due to olive oil’s anti-inflammatory and neuroprotective properties. Evidence from the Prevencion con Dieta Mediterranea (PREDIMED) trial indicates that a higher intake of olive oil combined with adherence to this diet may help protect against cognitive decline [103]. Moreover, higher olive oil intake was associated with a lower risk of dementia-related mortality, irrespective of diet quality [104]. In this context, the impact of both OLE and HT alone on cognitive health becomes intriguing. Yoon et al. investigated the effects of desert HT-enriched olive tree pearls (DOTPs) on cognitive function in healthy middle-aged and older individuals over 12 weeks using Cognitrax, a standardized cognitive assessment method [40]. They found significant improvements in complex attention in the DOTP group compared to the control and considerable time effects in various cognitive domains. Interestingly, the motor speed improved in the control group, possibly due to the olive oil in the placebo. However, DOTP consumption led to more pronounced time-dependent changes in cognitive functions compared to the control, suggesting the beneficial effects of high HT concentrations in DOTPs. The study also indicated that DOTPs were more effective in improving cognitive function in older adults than in middle-aged adults. Daily consumption of DOTPs, rich in HT, may help maintain and improve cognitive function in elderly Japanese individuals, potentially contributing to an extension of the healthy life expectancy.

The broader body of literature provides additional evidence supporting the positive impact of HT supplementation on cognitive function. For instance, consumption of olive oil, of which HT is an important part, has been linked to a reduced risk of mortality related to dementia. Specifically, consuming a minimum of 7 g/d olive oil was associated with a 28% decrease in the dementia-related risk [104]. Conversely, HT exhibited significant neuroprotective properties in numerous animal model studies, particularly in inhibiting the fibrillization of Tau protein and preventing β-amyloid aggregation, one of the causes of Alzheimer’s disease [105]. Moreover, HT treatment reversed deficits in spatial and working memory induced by Aβ1–42 oligomers. It prevented activation of apoptotic pathways [106] while improving spatial memory and reducing apoptosis in the cortex and hippocampus of the Alzheimer’s disease mouse model [107].

## 5. Limitations and Future Perspectives

**Figure 3:**
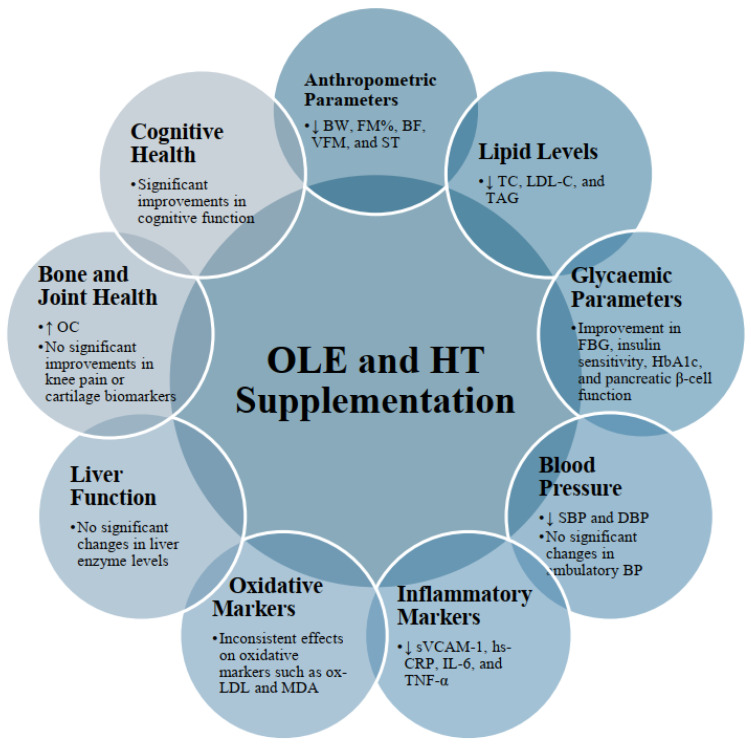
The positive impacts of oleuropein (OLE) and hydroxytyrosol (HT) as evidenced by human randomized controlled trials (RCTs). Body weight (BW), fatty mass percentage (FM%), body fat (BF), visceral fat mass (VFM), skinfold thickness (ST), total cholesterol (TC), low-density lipoprotein cholesterol (LDL-C), triacylglycerols (TAG), fasting blood glucose (FBG), glycated hemoglobin (HbA1c), systolic blood pressure (SBP), diastolic blood pressure (DBP), circulating vascular cell adhesion molecule-1 (sVCAM-1), high-sensitivity C-reactive protein (hs-CRP), interleukine-6 (IL-6), tumor necrosis factor-α (TNF-α), oxidized-LDL (ox-LDL), malondialdehyde (MDA), osteocalcin (OC), ↑—increased levels, ↓—decreased levels.

This systematic review summarized the beneficial effects of OLE and HT from several human RCTs (Figure 3), providing a new perspective compared to broader studies on olive oil consumption. This targeted approach revealed precise health benefits, covering glycemic control, blood pressure, inflammatory and oxidative markers, liver function, bone and joint health, and cognitive health, offering a comprehensive understanding of OLE’s and HT’s impacts. The research included diverse populations, integrating findings not only from human but also from animal studies for a robust perspective. By systematically reviewing existing studies, the article provided a thorough evidence analysis, considering the mechanisms of action of OLE and HT and acknowledging the study design variability. These insights have practical implications for public health, informing dietary recommendations, supplement use, and therapeutic strategies. 

However, some limitations of the review should be acknowledged. Firstly, the included studies were predominantly of a short duration (3–12 wk), which may limit the ability to observe long-term effects or trends in outcomes related to OLE and HT supplementation. These short durations could mask these compounds’ delayed or cumulative effects on health outcomes. Another area for improvement is the consistency in dosing protocols across the included studies. The variability in the dose of OLE and HT supplementation makes it challenging to determine the optimal dosage for achieving desired health outcomes. Without standardized dosing protocols, it becomes difficult to compare the efficacy of different interventions and draw robust conclusions about the effects of these compounds. 

Additionally, the included studies are demographically sparse, with a predominant focus on subjects with cardiometabolic diseases, such as overweight/obese individuals, those with T2DM, and pre-hypertensive subjects. This limited representation of diverse demographic groups may restrict the generalizability of the findings to broader populations. Including a more varied range of participants would provide a better understanding of how OLE and HT supplementation may impact various subgroups within the population.

Overall, all the studies included in the systematic review generally demonstrated improvements in the LDL-C levels but did not indicate any significant effects on the HDL-C concentrations. The European Food Safety Authority (EFSA) has concluded that sufficient evidence supports a cause-and-effect relationship between the consumption of olive oil phenols, standardized by the content of HT and its derivatives, and the protection of LDL-C particles from oxidative damage. To benefit from this effect, individuals should consume at least 5 mg of HT and its derivatives in olive oil daily [59]. The evidence is inconsistent regarding maintaining the average blood HDL-C concentrations, and there is no precise biological mechanism through which olive oil phenols could achieve this effect. As noted also by the EFSA, there is currently no established causal relationship between the consumption of phenols in olive products, standardized by HT content or its derivatives, and the maintenance of normal blood pressure. Moreover, beyond the observed decrease in the LDL-C levels, there is a lack of substantial evidence supporting any other significant health benefits associated with supplementation of OLE or HT. The reason for this might stem from the compounds’ bioavailability. OLE displayed greater stability during digestion than HT, reaching the colon unchanged and producing a wider variety of microbial metabolites [61]. However, when administered in liquid form, both compounds quickly reached peak concentrations, typically 30 min after intake [30]. Therefore, the quantity and form of OLE and HT could determine their beneficial effects in humans. Healthcare providers can combine OLE and HT supplementation with comprehensive lifestyle interventions to optimize cardiovascular health outcomes. Personalized supplementation plans based on individual health profiles and dietary habits could provide an opportunity to assess changes in lipid profiles, oxidative stress markers, and other relevant health parameters.

## 6. Conclusions

The Mediterranean diet, characterized by the consumption of olive oil rich in phenolic compounds like OLE and HT, has long been associated with reduced CVD risk and improved cardiometabolic health. The present systematic review investigated the effects of OLE and HT supplementation on various health parameters, including lipid levels, glycemic control, blood pressure, liver function, bone and joint health, and cognitive function. The review revealed mixed but promising results, with some studies showing significant reductions in body weight, TC, LDL-C, and TAG, and improvements in blood glucose levels and insulin sensitivity. However, the outcomes varied across different populations and intervention protocols, highlighting the need for further research to clarify the optimal dosing and duration of supplementation. Despite these mixed results, OLE and HT supplementation are potential interventions to enhance cardiometabolic, bone, joint, and cognitive health, although addressing study limitations such as the short duration and inconsistent dosing protocols is essential for fully understanding their therapeutic benefits. Therefore, future research should consider the bioavailability and the optimal dosage and duration of OLE and HT supplementation, as studies have demonstrated that their form of administration, dosage, absorption, metabolism, and tissue distribution vary. Considering OLE’s higher stability, it is recommended to use OLE as the preferred HT precursor for incorporation into foods or nutraceutical formulations due to its superior stability. Personalized supplementation plans, tailored to individual health profiles and dietary habits, can help monitor changes in lipid profiles, oxidative stress markers, and other health parameters.

## Figures and Tables

**Table 1:** The study’s rationality and inclusion criteria summarized under the PICOS framework.

Criteria	Descriptions
Population	Adult participants (aged > 18 years) regardless of their health status, with the exclusion of pregnant individuals.
Intervention	Standardized supplement, pure compounds, or enriched foods with OLE and/or HT as part of the intervention.
Comparison	Placebo that resembled the intervention but lacked the presence of OLE and/or HT.
Outcome	Evaluation of the effects of OLE and/or HT supplementation on systemic health outcomes, including but not limited to lipid markers (TC, LDL-C, HDL-C, TAG), glucose metabolism indicators (FBG, HbA1c), blood pressure measurements (SBP, DBP), inflammation markers (CRP, IL-6, etc.), liver function tests (ALT, AST, GGT), cognitive health tests, etc.
Study	RCT applying either a crossover or parallel trial design, with a duration of at least two weeks.

**Table 2:** The scoring criteria for the modified Jadad score.

Items of Modified Jadad Score	Scoring Criteria	Points
Randomization	Proper randomization described	2
	Study described as randomized but without proper randomization	1
	Randomization absent or inappropriate	0
Concealment of allocation	Proper allocation concealment described	2
	Study mentioned using an allocation concealment method but without proper description	1
	Allocation concealment method not described	0
Blinding	Proper blinding with identical placebo/control	2
	Inadequate or undisclosed blinding	1
	Double blinding inappropriate or absent	0
Withdrawals and dropouts	Numbers and reasons for withdrawals and dropouts detailed	1
	Numbers and reasons for withdrawals and dropouts not detailed	0
Total score	Sum of scores across all components	0–7

**Table 3:** Characteristics of included randomized controlled trials (RCTs) evaluating diverse health outcomes of oleuropein (OLE) and hydroxytyrosol (HT) supplements or enriched foods among various demographics.

Study (Year), Country	Study Design	Participants	Type of Intervention	Intervention/d	Control	Duration	Total Sample (I/C)	Measured Outcomes
Vázquez-Velasco et al. (2011), Spain [35]	Randomized, single-blinded, placebo-controlled, crossover trial	Healthy subjects	ESO	10–15 g/d of HT-ESO (45–50 mg HT)	10–15 g/d of control sun- flower oil	3 wk	22 (11/11)	CVD risk factors: Lipid profile, inflammatory markers
de Bock et al. (2013), New Zealand [44]	Randomized, double-blinded, placebo-controlled, crossover trial	Overweight subjects	Pure compounds	51.1 mg/d OLE and 9.7 mg/d HT	Placebo	12 wk	45 (23/22)	CVD risk factors: Insulin sensitivity, lipid profile, hormones, adiposity, blood pressure, liver parameters
Filip et al. (2015), Poland [41]	Randomized, double-blinded, placebo-controlled, parallel trial	Postmenopausal and osteopenic women	Standardized supplement	250 mg/d olive extract (100 mg OLE) and 1000 mg Ca	Placebo and 1000 mg Ca	12 m	64 (32/32)	Bone metabolism and CVD risk factors: Bone health, lipid profile, inflammatory markers
Colica et al. (2017), Italy [36]	Randomized, double-blinded, placebo-controlled, crossover trial	Healthy subjects	Pure compound	15 mg/d HT	Placebo	3 wk	28 (14/14)	Antioxidant and metabolic state: Body composition, antioxidant status, glucose metabolism, lipid profile, gene expression
Lockyer et al. (2017), New Zealand [45]	Randomized, double-blinded, placebo-controlled, crossover trial	Pre-hypertensive subjects	Pure compounds	136.2 mg/d OLE and 6.4 mg/d HT	Placebo	6 wk	60 (30/30)	CVD risk factors: Blood pressure, lipid profile, glucose metabolism, inflammatory markers
Araki et al. (2019), Japan [46]	Randomized, unblinded, placebo-controlled, parallel trial	Prediabetic subjects	OLI	990 mL/d OLI (320.8 mg OLE and 11.9 mg HT)	990 mL/d OLI (23.8 mg OLE and 3 mg HT)	12 wk	57 (28/29)	CVD risk factors: Anthropometric parameters, glucose metabolism, lipid profile
Stevens et al. (2021), the Netherlands [42]	Randomized, double-blinded, placebo-controlled, parallel trial	Overweight/obese subjects	Standardized supplement	500 mg/d olive leaf extract (83.5 mg OLE)	Placebo	8 wk	77 (39/38)	CVD risk factors: Anthropometric parameters, lipid profile, blood pressure, glucose metabolism, liver function
Fytili et al. (2022), Greece [37]	Randomized, double-blinded, placebo-controlled, parallel trial	Overweight/obese subjects	Pure compound	5 or 15 mg/d HT	Placebo	6 m	29 (18/11)	CVD risk factors: Anthropometric parameters
Horcajada et al. (2022), Belgium [43]	Randomized, double-blinded, placebo-controlled, parallel trial	Subjects with knee pain	Standardized supplement	250 mg/d olive leaf extract (100 mg OLE)	Placebo	6 m	124 (62/62)	Joint functional capacity: Joint health, cartilage degradation, inflammatory markers
Ikonomidis et al. (2023), Greece [38]	Randomized, double-blinded, placebo-controlled, crossover trial	Chronic coronary artery syndrome subjects	Standardized supplement	1650 mg olive oil and 10 mg/d HT	1650 mg olive oil	4 wk	30 (17/13)	CCD: Vascular function, oxidative stress, inflammatory markers, lipid profile
Binou et al. (2023), Greece [39]	Randomized, single-blinded, placebo-controlled, parallel trial	Overweight/obese and T2DM subjects	Enriched WWB	60 g/d HT-enriched WWB (32.5 mg HT)	60 g/d conventional WWBd	12 wk	75 (38/37)	CVD risk factors: Anthropometric parameters, glucose metabolism, blood pressure, lipid profile, liver parameters, inflammatory markers, hormones
Yoon et al. (2023), Japan [40]	Randomized, double-blinded, placebo-controlled, parallel trial	Healthy middle-aged and older subjects	Enriched DOTPs	6 g/d HT-enriched DOTPs (97.2 HT)	6 g/d DOTPs	12 wk	72 (36/36)	Cognitive function: Memory, attention, processing speed, executive function, reaction time, and motor skills

Abbreviations: intervention/control (I/C), cardiovascular disease (CVD), type 2 diabetes mellitus (T2DM), chronic coronary disease (CCD), calcium (Ca), enriched sunflower oil (ESO), olive leaf infusion (OLI), whole wheat bread (WWB), desert olive tree pearls (DOTPs), weeks (wk), months (m).

**Table 4:** Risk of bias assessment of the studies included in this systematic review using the modified Jadad score.

Study	Items of Modified Jadad Score				
	Randomization	Concealment of Allocation	Double-Blinding	Withdrawals and Dropouts	Total Score
Vázquez-Velasco et al. (2011) [35]	2	0	2	1	5
de Bock et al. (2013) [44]	2	2	2	1	7
Filip et al. (2015) [41]	2	0	2	1	5
Colica et al. (2017) [36]	2	0	2	1	5
Lockyer et al. (2017) [45]	2	2	2	1	7
Araki et al. (2019) [46]	1	0	0	1	2
Stevens et al. (2021) [42]	2	2	2	1	7
Fytili et al. (2022) [37]	1	2	2	1	6
Horcajada et al. (2022) [43]	2	0	2	1	5
Ikonomidis et al. (2023) [38]	2	2	2	1	7
Binou et al. (2023) [39]	2	0	1	1	4
Yoon et al. (2023) [40]	2	1	2	1	6

## Data Availability

The original contributions presented in this study are included in the article; further inquiries can be directed to the corresponding author/s.
